# Supplementary figures and images for: Optimization of Aspergillus versicolor Culture and Aerosolization in a Murine Model of Inhalational Fungal Exposure
Source: J Fungi (Basel). 2023 Nov 8;9(11):1090. doi: 10.3390/jof9111090 (PMC10672600; doi:10.3390/jof9111090)

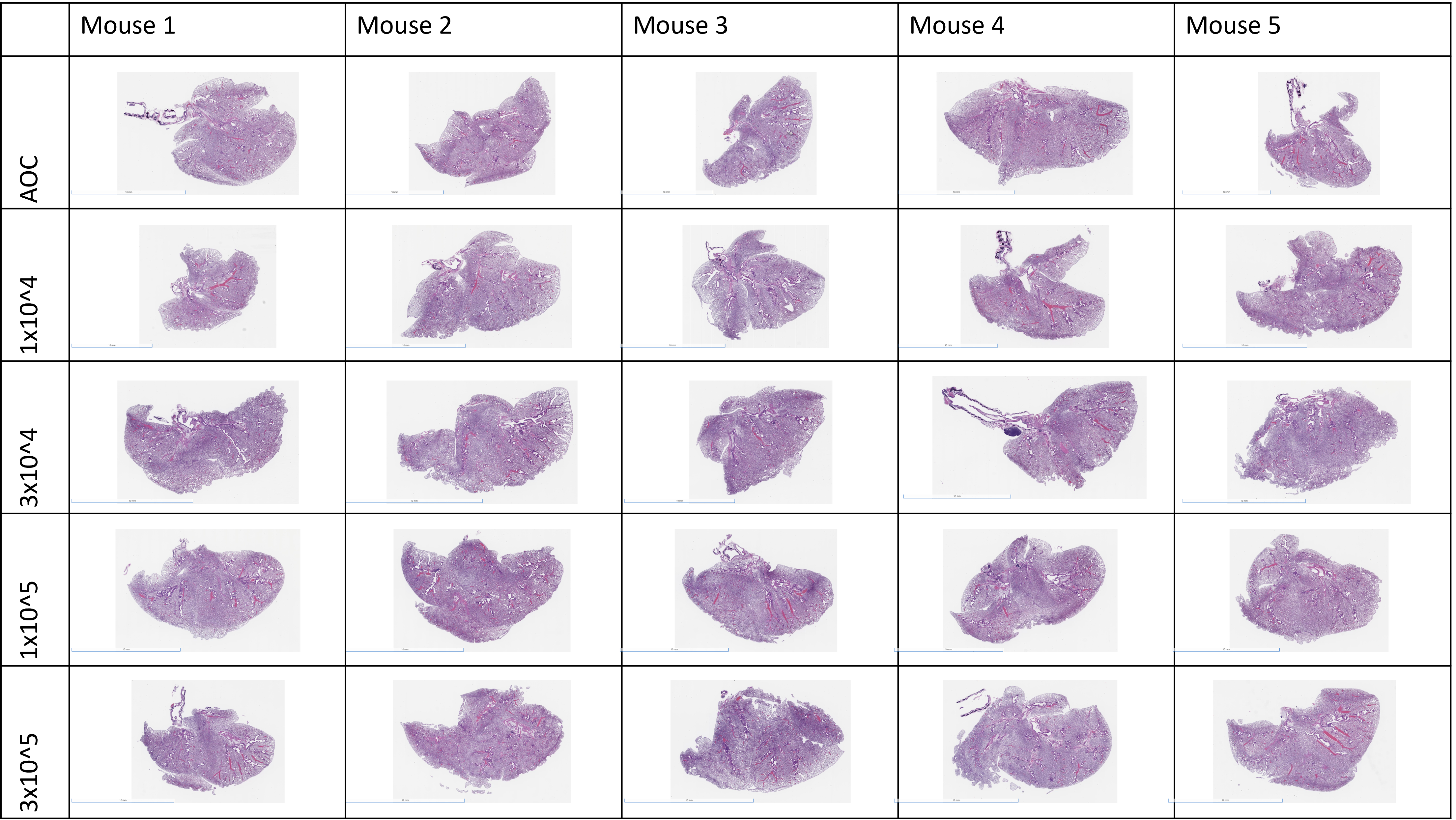

Supplement: Supplementary file 1 [file jof-09-01090-s001.zip › Figure S1.tif]

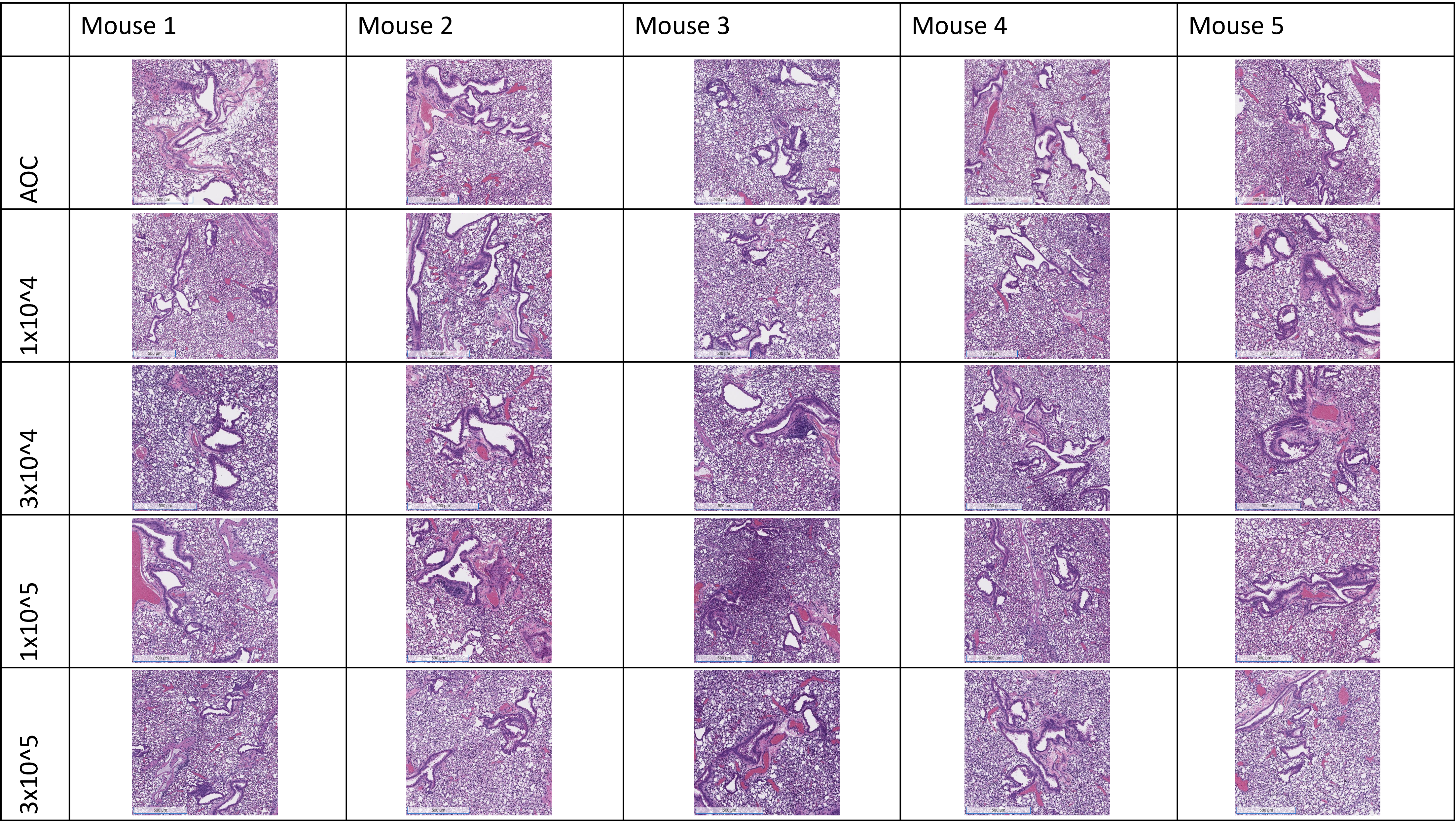

Supplement: Supplementary file 1 [file jof-09-01090-s001.zip › Figure S2.tif]

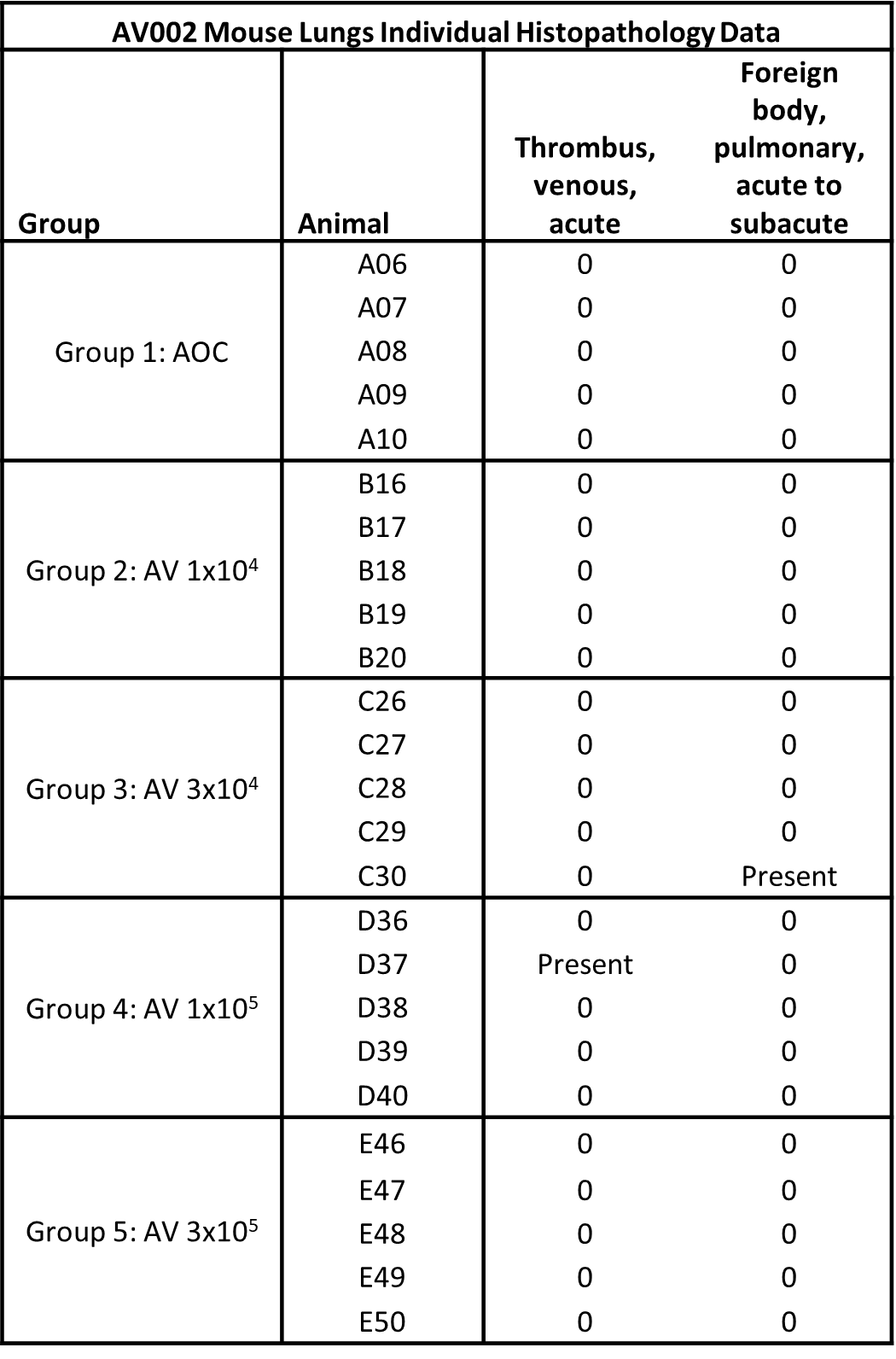

Supplement: Supplementary file 1 [file jof-09-01090-s001.zip › Table S1.tif]
